# Central Illinois Dental Society

**Published:** 1883-03

**Authors:** 


### Central Illinois Dental Society.

A number of dentists met at Streator, Ills., in July, 1882, and organized "The Central Illinois Dental Society," with Dr. J. Frank Marriner, of Ottawa, as President, and Dr. C. R. Taylor, of Streator, as Secretary.

Two meetings will be held each year; the annual meeting on the second Tuesday of July, and a semi-annual meeting on the second Tuesday of January.

The first semi-annual meeting was held at Wenona, Tuesday, January 9th.

Dr. Garrett Newkirk, read a paper on "The Ideal Dental Office," which was full of practical suggestions, and elicited quite a lively discussion. He took the ground that the dentist should strive to secure his rooms while being built, and have them arranged for this special purpose. He favored a northeastern light, and for sanitary reasons would eschew paper on the walls, and carpet on the floor; would have the laboratory arranged in two rooms, one with outside openings to be used for vulcanizing, grinding on the lathe, and similar gas and dust producing operations.

Dr. J. Frank Marriner read a paper entitled "Random Thoughts." It was a plea for the adornment of our offices, and method in our work, such as recording cases, etc., with a description of some delicate instruments for treating nerve canals and removing tartar.

He believed the best treatment for the "so-called Riggs disease," to be a thorough removal of tartar. No one form of instruments would answer for all cases, and he insisted on the dentist being able to make his own instruments.

To show that any one could do it, he exhibited some scalers of a very delicate pattern, and some nerve broaches shaped from piano wire, all of his own make, and described his process of forming, tempering, etc.

Dr. C. R. Taylor read a paper on "Inflammation," illustrating it by diagrams showing the effect of inflammation upon the circulation, etc. The discussion of the paper turned somewhat upon the systemic instead of local treatment of sensitive dentine.

The society adjourned to meet in El Paso, July 10th, 1883.

